# Risk factors associated with albuminuria in Rwanda: results from a STEPS survey

**DOI:** 10.1186/s12882-021-02574-w

**Published:** 2021-11-01

**Authors:** Candide Tran Ngoc, Prebo Barango, Roger Harrison, Andrew Jones, Steven Velabo Shongwe, Albert Tuyishime, François Uwinkindi, Hongyi Xu, Stephanie Shoop-Worrall

**Affiliations:** 1 World Health Organization, Country Office for Rwanda, Kigali, Rwanda; 2 World Health Organization, Inter Country Support Team, Eastern and Southern Africa, Harare, Zimbabwe; 3 Division of Population Health, Health Services Research & Primary Care, The University of Manchester, Manchester, UK; 4 World Health Organization, Regional Office for Africa, Brazzaville, Republic of Congo; 5 Rwanda Biomedical Center, Kigali, Rwanda; 6 World Health Organization, Headquarters, Geneva, Switzerland; 7 Division of Musculoskeletal and Dermatological Sciences, The University of Manchester, Manchester, UK

## Abstract

**Background:**

Non-communicable diseases (NCDs) are a growing burden which affects every part of the world, including developing countries. Chronic kidney disease (CKD) has varied etiology which can result from or complicate other NCDs such as diabetes and cardiovascular diseases. The growing prevalence of NCDs coupled with the increasing age in most developing countries, has seen a marked increase of CKD in these settings. CKD has been described as “the most neglected NCD” and greatly affects the quality of life of patients. It also places a huge economic burden on societies. However, few epidemiological data exist, particularly in sub-Saharan Africa. Assessment of the prevalence of albuminuria as a marker of kidney damage and CKD progression and its main risk factors was thus needed in Rwanda.

**Methods:**

This study analyzed data collected during the first STEPwise approach to NCD risk factor Surveillance (STEPS) survey in Rwanda, conducted from 2012 to 2013, to assess the prevalence of albuminuria. A multistage cluster sampling allowed to select a representative sample of the general population. Furthermore, descriptive, as well as univariable analyses and multiple logistic regression were performed to respond to the research question.

**Results:**

This survey brought a representative sample of 6,998 participants, among which 4,384 (62.65%) were female. Median age was 33 years (interquartile range, IQR 26-44), and over three quarters (78.45%) lived in rural areas. The albuminuria prevalence was 105.9 per 1,000 population. Overall, semi-urban and urban residency were associated with lower odds of CKD (odds ratio, OR 0.36, CI 0.23-0.56, *p*<0.001 and OR 0.34, CI 0.23-0.50, *p*<0.001, respectively) than rural status. Being married or living with a partner had higher odds (OR 1.44 (CI 1.03-2.02, *p*=0.031) and OR 1.62 (CI 1.06-2.48, *p*=0.026), respectively) of CKD than being single. Odds of positive albuminuria were also greater among participants living with human immunodeficiency virus (HIV) (OR 1.64, CI 1.09- 2.47, *p*=0.018). Gender, age group, smoking status and vegetable consumption, body mass index (BMI) and hypertension were not associated with albuminuria.

**Conclusion:**

The albuminuria prevalence was estimated at 105.9 per 1,000 in Rwanda. Rural residence, partnered status and HIV positivity were identified as main risk factors for albuminuria. Increased early screening of albuminuria to prevent CKD among high-risk groups, especially HIV patients, is therefore recommended.

**Supplementary Information:**

The online version contains supplementary material available at 10.1186/s12882-021-02574-w.

## Introduction

Chronic kidney disease (CKD) affects approximately 10 to 15% of the global population [1], and has been described as the most neglected NCD [2]. Early-stage CKD is rarely detected [3, 4] and can go unnoticed until 30 to 40% of the kidney function is lost [5]. Early signs and symptoms of disease include tiredness, lack of appetite, itching, and changes in urination [6–8], as well as uremic symptoms and sexual dysfunction [9–12]. The condition worsens with cramps, vomiting, back pain, and bloody urine [6, 7] and is usually diagnosed at a late stage [13, 14]. CKD is defined by a reduced renal glomerular filtration rate (GFR) inferior to 60 mL/min per 1·73 m^2^ [12], and/or markers of kidney damage for over three months, usually marked by albuminuria [15].

Without successful treatment, CKD can progress to end-stage renal disease (ESRD), corresponding to a GFR less than 15 mL/min per 1·73m^2^ and total kidney failure [12]. As the disease progresses, major complications of CKD include hypertension, anemia, bone and electrolytes disorders and volume retention [9, 10]. All of these greatly affect the quality of life of patients, and are linked to high mortality levels [12]. Low detection and severe complications in late-stage disease has meant that CKD figures among the three causes of death with the greatest increase from 1990 to 2015 globally [16], in contrast with other non-communicable diseases (NCDs) [16].

In low-income countries, the silent growing CKD epidemic [17, 18] places both a huge health and financial burden on the individuals and the society [2, 19, 20]. While management of CKD involves controlling its associated cause(s) and complications [21, 22], ESRD requires more aggressive, financially costly therapies such as renal replacement therapy (RRT) and/or kidney transplant [21, 22]. A lack of early detection, coupled with limited access to RRT for ESRD in fragile healthcare systems [23] amplifies the impact of the disease [1, 23, 24]. When detected early [4], available and affordable therapies can slow or prevent progression of CKD to ESRD [25]. Identification and screening of at-risk individuals is therefore critical [26] to reduce the risk of the disease, particularly in resource-limited settings [27, 28]. However, knowledge of risk factors for CKD, particularly on the African continent, is limited.

Although little information on CKD is available on the African continent [29–31]; recent studies estimated its prevalence in 21 countries in sub-Saharan Africa between 10.1 to 15.8% [30–34]

Some CKD risk factors are well-recognized globally, such as diabetes, hypertension and ageing [35], with diabetes being the leading cause of CKD globally [26]. However, others such as smoking and obesity may be more relevant within certain parts of the globe. Changes in lifestyles led to an increase of such risk factors in all countries of the African region, resulting in an upsurge in CKD in the region [36]. Potential additional risk factors for CKD include socio-economic status, genetic factors, infections, medicines, and environmental factors [19, 26, 37–41]. In addition to these factors, many risk factors for CKD are still unknown, especially in resource-limited countries [29]. There is thus a need to explore the extent to which this applies to other populations [32].

The World Health Organization (WHO)’s STEPS (STEPwise approach to NCD risk factor Surveillance) instrument [42] collects and analyzes information on main NCDs risk factors in a standardized way [43]. It collects information on four main behavioral NCD risk factors, namely tobacco use, alcohol consumption, diet and physical activity [44]. It also gathers evidence on four major biological risk factors for NCDs: overweight/obesity, increased blood pressure, raised blood glucose and raised cholesterol [44].

In view of the above-mentioned challenges, it was thus deemed relevant to estimate the prevalence of albuminuria as a marker of kidney damage and CKD progression [15] in Rwanda, as well as to investigate its risk factors in the country to allow an earlier detection of the at-risk people; and to inform policy-makers for improved planning. This could be comprehensively assessed using the standardized WHO STEPS approach. The main objective of this study was therefore to assess the main risk factors associated with albuminuria in Rwanda.

## Methods

### Study population

**Fig. 1:**
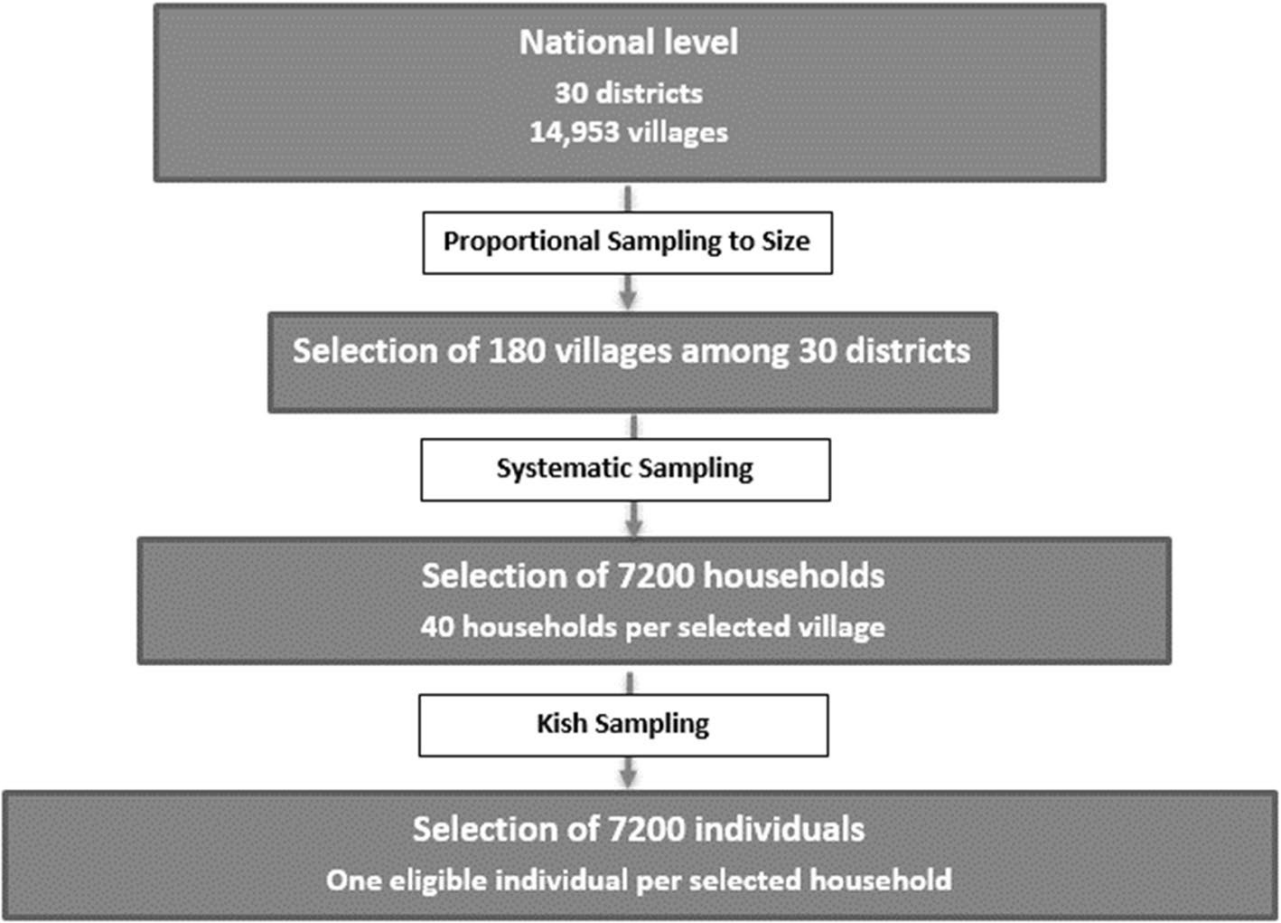
Sampling flow chart

This analysis included participants recruited to the STEPS survey, a cross-sectional population-based study of NCD risk factors in Rwanda. Data collection took place between November 2012 to March 2013 [34]. The STEPS survey aimed to identify risk factors for albuminuria used a representative sample of the Rwandan population using three stage cluster sampling [44]. The overall sample size necessary for the study was deemed to be 7200 participants over ten age and sex groups, considering a 50% prevalence of risk factors, since no previous data were available, and assuming a 20% non-response rate, a 1.5 design effect to account for complex sample design, which represents the likelihood for a study participant to present the risk factor of interest and is the recommended value for most STEPS surveys [44]; 5% margin of error and 95% level of confidence [44]. Participants were randomly chosen using multistage cluster sampling [45] (Fig. 1) from enumeration areas (Eas) previously defined during the 2012 census [34] and corresponding to villages [44, 46]. A maximum of one eligible individual among each of the households was randomly selected (Fig. 1). Using the Kish method [44, 47].

Inclusion criteria included: individuals within selected households aged between 15 and 64 years. Furthermore, pregnant women were excluded from height and weight measurements [44].

### Data collection

Data collection was conducted by sixteen teams, each composed of three data collectors, one laboratory technician and one supervisor [34].

The Rwanda NCD risk factor survey used the WHO’s three-step approach for surveillance of risk factors for NCDs [34], which involved:

i) Step 1: A behavioral risk factors interview through a questionnaire, which was completed for participants at their domicile for confidentiality purposes on the first day of the data collection.
ii) Step 2: Physical measurements, which took place the same day as Step 1.
iii) Step 3: Biological measurements, which took place the second day after participants had received fasting instructions on day 1.

The questionnaire included questions on behavioral risk factors for NCDs and physical and biological measurements.

For Step 1, behavioral risk factors were surveyed by data collectors using electronic portable devices such as personal digital assistants (PDAs). Demographic factors included age, sex, marriage status (single, cohabitating, married, separated, divorced, widowed residence (rural/urban/semi-urban), level of education (no formal schooling, complete primary, complete secondary and at least undergraduate university schooling completed) and employment status (employee, self-employed, student, non-paid work, retired, unemployed). Lifestyle factors included tobacco (tobacco consumption during the past 12 months ) and alcohol use (alcohol consumption during the past 30 days), fruits and vegetables consumption (number of servings per day) and physical activity (number of days in a week with moderate physical activity ) [34].

For Step 2, blood pressure, weight and height were measured using the following devices: OMRON M4 Digital Automatic Blood Pressure Monitors; Genesis growth scales; and Figure Finder® constant tension measuring tapes, respectively [34]. Weight and height were measured to the nearest 0.1 kg and to the nearest whole centimeter, respectively and both were used to calculate body mass index (BMI) (underweight, normal, overweight and obese). Systolic and diastolic blood pressure was measured three times [34]; and an average of the three results was taken [34]. Hypertension was defined as a systolic blood pressure equal to or above 140 mmHg and/or diastolic blood pressure equal to or above 90 mmHg. Participants receiving antihypertensive drugs were included into the survey.

In step 3, participants fasted from the evening of the previous day (from 10 PM) until measurements were made in the following morning [34]. During step 3, capillary blood samples were taken using the finger prick method [48], and total cholesterol (mmol/L or mg/dl) and fasting blood glucose (mmol/L) were assessed using CardioChek PA devices [49] while human immunodeficiency virus (HIV) status was conducted using an antibody-based method. For every tenth finger-prick test, a sample of venous blood was also taken at the National Reference Laboratory for counter verification [34]. Urine samples were collected on day 1 in a non-fasting state as per WHO’s guidance [44]. Raised fasting blood glucose was defined as fasting capillary blood glucose ≥ 6.1 mmol/L (110 mg/dl). Furthermore, participants receiving an antidiabetic treatment were included into this study. Raised total cholesterol was defined as ≥ 5.0 mmol/L or ≥ 190 mg/dl.

### Outcome measurement

Albuminuria was rated as positive if estimated at above 30 mg/dl using dipsticks [50]. Results were then confirmed using COBAS lab analyzers [51]; and normal laboratory values for urine albumin were 0 – 8 mg/dl [46]. This study therefore considered only the participants who consented to take part into step 3 and for whom albuminuria results were available. Participants with abnormal test results were then referred to the nearest health facility for further investigations and proper management of their condition if necessary [46].

### Statistical analysis

#### Estimating albuminuria prevalence

Albuminuria prevalence was estimated by dividing the number of subjects with albuminuria by the total number of participants who agreed to provide urine samples. This figure was then multiplied by 10 to calculate the prevalence per 1,000 people; and results displayed by sex.

#### Assessing risk factors for albuminuria

Initially, associations were assessed between each risk factor and albuminuria using a Chi-squared test.

Multicollinearity between continuous variables was then tested using Spearman’s correlation and variables with greater data availability were preferred from the initial demographic, biological and risk factors variables for entry to a multivariable model where significant correlations arose [52].

Furthermore, variables of clinical interest that were correlated with variables in the primary multivariable model were additionally explored as risk factors for albuminuria in secondary analyses (Supplementary materials).

All analyses were undertaken in STATA 15 (StataCorp 2017) [53].

## Results

### Study response rate and Power Analysis

Overall, 7,240 respondents consented to take part in the first step of this study.

Among the initial respondents, 7,224 (99.78%) participated in Step 2 (physical measurements), while 7,161 (98.91%) consented to take part in the final step (biological measurements). Results for albuminuria were available for 6,998 (96.66%) participants, who comprised the final study population.

The required sample size was estimated at n=6549 to detect an odds ratio of at least 1.5, or n=6350 to detect an odds ratio lower or equal to 0.6, for any factor with an estimated prevalence of 10%, 95% confidence level, 90% precision and with a 1:10 ratio of people with albuminuria to those without albuminuria.

### Demographic, biological and lifestyle characteristics of participants

As shown in Table 1, 4,384 (62.65%) participants were females. Median age was 33 years (IQR 26-44). Furthermore, most of the participants (78.45%) lived in rural areas. Most of the group (60.40%) had no formal education; 82.09% of the participants were self-employed, and most were married (56.91%).

**Table 1:** Demographic, biological and lifestyle characteristics of participants

	No. of patients with available data (%)	N (%) or Mean (SD) or Median (IQR)
**Socio-demographic characteristics**		
**Sex**	**6998 (100.00)**	
Male		2614 (37.35)
Female		4384 (62.65)
**Age group**	**6993 (99.93)**	
15-24		1460 (20.88)
25-34		2306 (32.98)
35-44		1513 (21.64)
45-54		1020 (14.59)
55-64		694 (9.92)
**Residence**	**6998 (100.00)**	
Rural		5490 (78.45)
Urban		921 (13.16)
Semi-urban		587 (8.39)
**Educational level**	**6988 (99.86)**	
No formal education		4223 (60.40)
Completed primary		2458 (35.17)
Completed secondary		200 (2.86)
Completed at least undergraduate university		107 (1.53)
**Employment status**	**6983 (99.79)**	
Employee		210 (3.01)
Self-employed		5732 (82.09)
Student		480 (6.87)
Non-paid work		232 (3.32)
Retired		8 (0.11)
Unemployed		321 (4.60)
**Marital status**	**6986 (99.83)**	
Single		1647 (23.58)
Cohabitating		493 (7.06)
Married		3976 (56.91)
Separated		226 (3.24)
Divorced		143 (2.05)
Widowed		501 (7.17)
**Physical and biological measurements**		
**Body Mass Index (BMI) (kg/m2)**	**6978 (99.71)**	**22.10 (20.41-24.17)**
Underweight (<18.5)		518 (7.42)
Normal (<=18.5 and =<24.9)		5202 (74.55)
Overweight (>=25.0 and =<29.9)		1017 (14.57)
Obese (BMI>=30.0)		241 (3.45)
**Hypertension status**	**6998 (100.00)**	
Normal BP (systolic BP <140 mmHg and diastolic BP <90 mmHg)		5757 (82.27)
Elevated BP (systolic BP>= 140 mmHg and/or diastolic BP>= 90 mmHg)		1241 (17.73)
**Cholesterol status**	**6973 (99.64)**	
Normal blood cholesterol (<5 mmol/L)		6756 (96.89)
Raised blood cholesterol (=>5mmol/L)		217 (3.11)
**Diabetic status**	**6629 (94.73)**	
Absence of diabetes (capillary blood glucose <5.6 mmol/L)		6308 (95.16)
Impaired fasting glycaemia (capillary blood glucose >=5.6mmol/L and <6.1 mmol/L)		110 (1.66)
Raised fasting blood glucose (capillary blood glucose >=6.1 mmol/L)		211 (3.18)
**Results of HIV test**	**5519 (78.87)**	
Positive		228 (4.13)
Negative		5291 (95.87)
**Risk factors for NCDs**		
**Tobacco use in the past 12 months**	**6991 (99.90)**	
Yes		1023 (14.63)
No		5968 (85.37)
**Alcohol use during the past 30 days**	**3327 (47.54)**	
Yes		2965 (89.12)
No		362 (10.88)
**Number of serving of fruit per day**	**4469 (63.86)**	**1 (1-1)**
1		3511 (78.56)
2		841 (18.82)
3 and over		117 (2.62)
**Number of serving of vegetables per day**	**6672 (95.34)**	**2 (1-2)**
1		3235 (48.49)
2		3311 (49.63)
3 and over		126 (1.89)
**Number of days in a week with moderate physical activity**	**3260 (46.58)**	**5 (3-6)**
1		170 (5.37)
2		375 (11.50)
3		443 (13.59)
4		239 (7.33)
5		448 (13.74)
6		1028 (31.53)
7		557 (17.09)

Most of participants (89.12%) reported alcohol consumption during the past 30 days and 14.63% of the survey participants had smoked any tobacco product in the past 12 months. Most subjects (78.56%) reported daily consumption of one serving of fruit, while 48.49 and 49.63% consumed between one to two servings of vegetables per day, respectively. Moreover, 1028 (31.53%) participants practiced a moderate physical activity six days in a week.

Three quarters (74.55%) of subjects had a normal BMI. Most of study participants (82.27%) had a normal BP. Moreover, 96.89% of subjects had a normal blood cholesterol. 95.26% of study subjects had a normal fasting blood glucose. Additionally, 4.13% were found positive for HIV.

Additionally, the proportion of selected socio-demographic, biological and behavioral characteristics of participants according to their marital status and residence is further described in the Supplementary materials. In general, older age groups, increased BMI as well as tobacco and alcohol use were more represented among married persons. This group also showed a greater proportion of participants with a higher vegetable consumption and moderate physical activity. Moreover, older age group, tobacco and alcohol use were more represented among participants living in a rural area (Supplementary materials).

### Albuminuria prevalence among study participants

Overall, 741 out of 6,998 (10.59%) of participants were found positive for albuminuria, which corresponds to a prevalence of 105.9 per 1,000 population.

Furthermore, albuminuria prevalence among females was almost twice than among males, with 287 (10.98%) out of 2,614 of males and 454 (19.36%) of 4,384 females presenting a positive albuminuria test result.

### Univariable associations with albuminuria

**Fig. 2:**
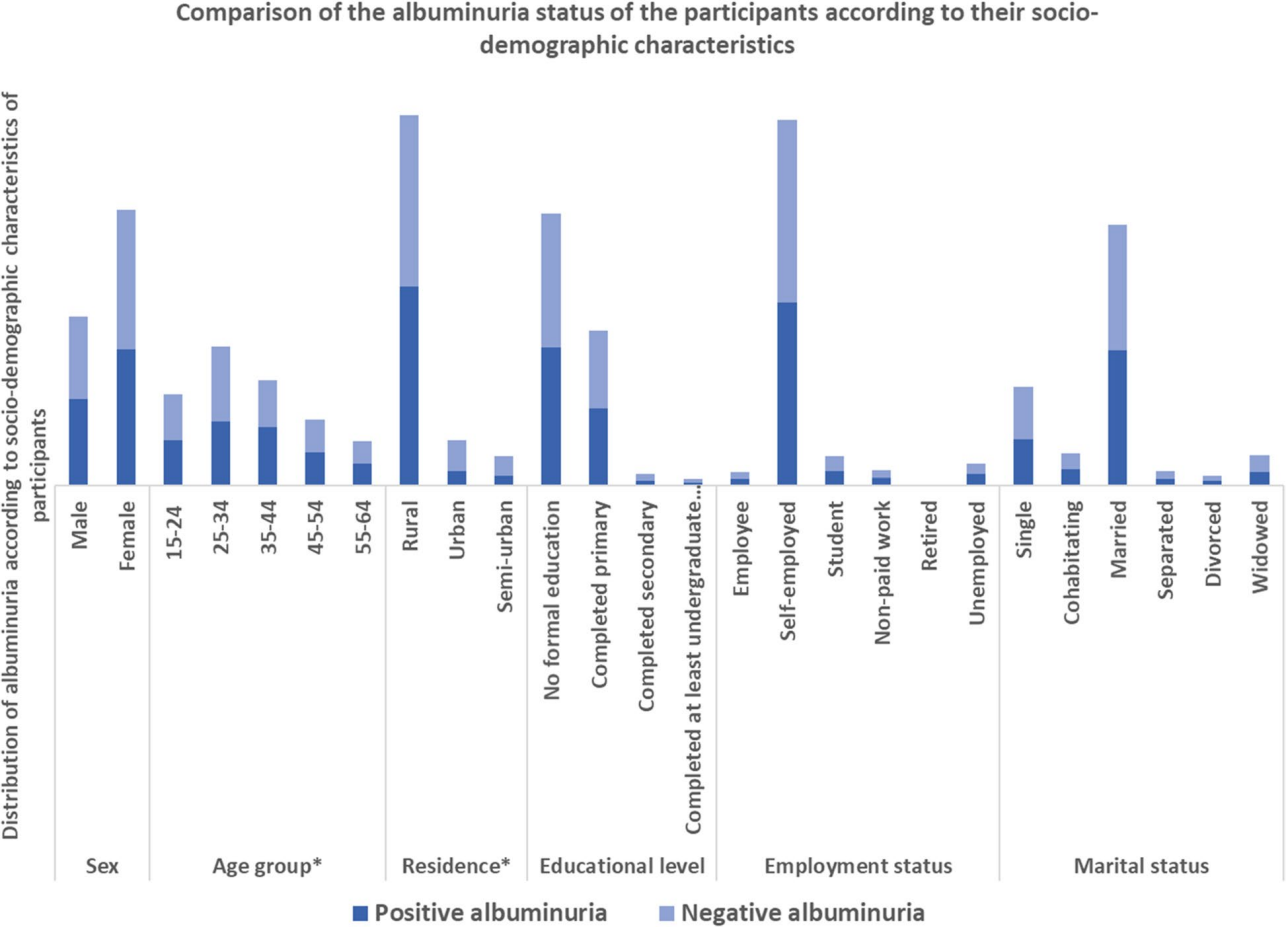
Comparison of the albuminuria status of the participants according to their socio-demographic characteristics. * The asterisk indicates variables that were significantly associated with albuminuria during the univariable analysis

**Fig. 3:**
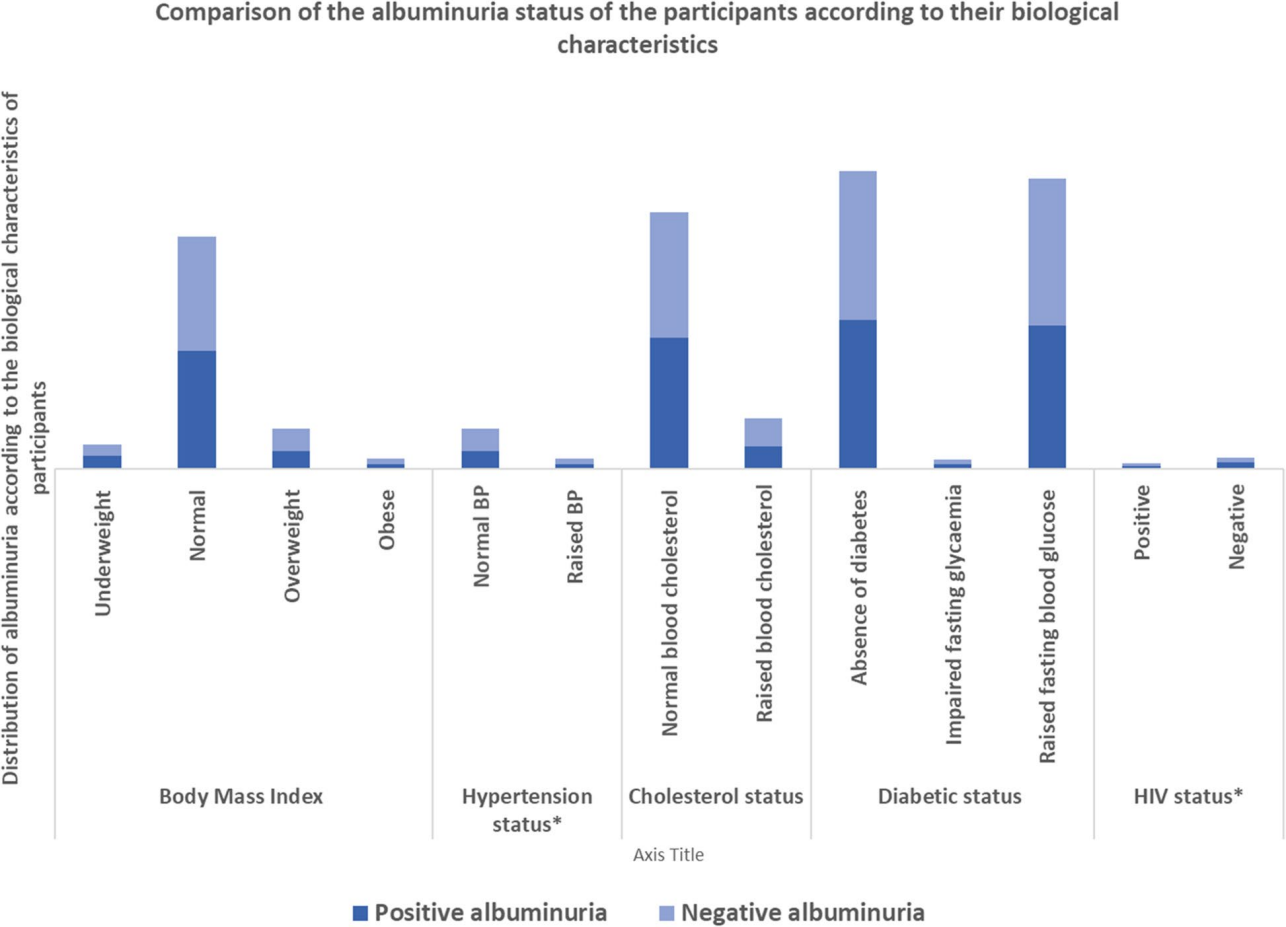
Comparison of the albuminuria status of the participants according to their biological characteristics.* The asterisk indicates variables that were significantly associated with albuminuria during the univariable analysis

**Fig. 4:**
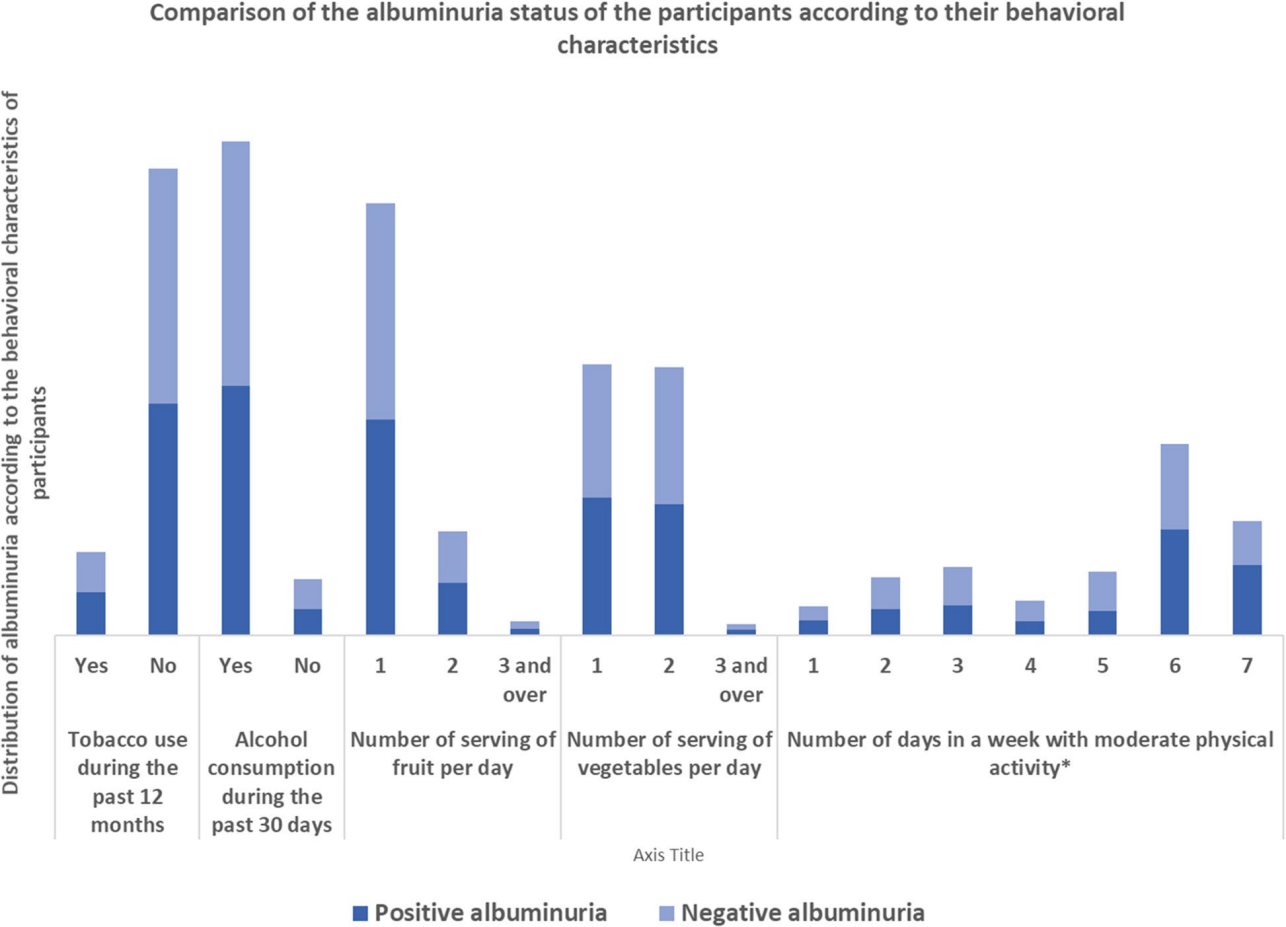
Comparison of the albuminuria status of the participants according to their behavioral characteristics. * The asterisk indicates variables that were significantly associated with albuminuria during the univariable analysis

In univariable analysis, there were significant differences in age group, residence, hypertensive and HIV status and levels of physical activity between people with and without albuminuria (Figs. 2, 3 and 4 and fifth column of Table 2).

**Table 2:** Results of the univariable and multivariable analyses

Variable	Variable category	Urine albumin		Univariable ***p***-value	Multivariable OR 95% confidence interval (CI)
		Positive N (%)	Negative N (%)		
**Socio-demographic characteristics**					
Sex	Male	287 (38.73)	2327 (37.19)	0.412	Reference
	Female	454 (61.27)	3930 (62.81)		0.99 (0.81-1.21)
Age group	15-24	149 (20.11)	1311 (20.97)	**0.017**	Reference
	25-34	214 (28.88)	2092 (33.46)		0.87 (0.62-1.20)
	35-44	193 (26.05)	1320 (21.11)		1.17 (0.83-1.66)
	45-54	111 (14.98)	909 (14.54)		0.92 (0.62-1.38)
	55-64	74 (9.99)	620 (9.92)		0.98 (0.61-1.58)
Residence	Rural	662 (89.34)	4828 (77.16)	**< 0.001**	Reference
	Urban	48 (6.48)	873 (13.95)		**0.34 (0.23-0.50)**
	Semi-urban	31 (4.18)	556 (8.89)		**0.36 (0.23-0.56)**
Educational level	No formal education	459 (61.94)	3764 (60.25)	0.484	Reference
	Completed primary	256 (34.55)	2202 (35.25)		1.1 (0.90-1.34)
	Completed secondary	15 (2.02)	185 (2.96)		0.96 (0.52-1.79)
	Completed at least undergraduate university	11 (1.48)	96 (1.54)		1.15 (0.48-2.77)
Employment status	Employee	21 (2.84)	189 (3.03)	0.918	Reference
	Self-employed	607 (82.14)	5125 (82.08)		0.95 (0.52-1.72)
	Student	46 (6.22)	434 (6.95)		1.35 (0.62-2.91)
	Non-paid work	25 (3.38)	207 (3.32)		1.05 (0.47-2.35)
	Retired	1 (0.14)	7 (0.11)		3.8 (0.36-40.48)
	Unemployed	39 (5.28)	282 (4.52)		1.82 (0.92-3.59)
Marital status	Single	152 (20.57)	1495 (23.93)	0.202	Reference
	Cohabitating	53 (7.17)	440 (7.04)		**1.62 (1.06-2.47)**
	Married	450 (60.89)	3526 (56.44)		**1.44 (1.03-2.02)**
	Separated	22 (2.98)	204 (3.27)		1.27 (0.69-2.33)
	Divorced	17 (2.30)	126 (2.02)		1.63 (0.83-3.19)
	Widowed	45 (6.09)	456 (7.30)		1.26 (0.75-2.09)
**Biological risk factors for CKD**					
Body mass index	Normal	570 (76.92)	4632 (74.27)	0.053	Reference
	Underweight	63 (8.50)	455 (7.30)		1.03 (0.71- 1.49)
	Overweight	85 (11.47)	932 (14.94)		0.83 (0.63- 1.08)
	Obese	23 (3.10)	218 (3.50)		1.15 (0.68- 1.93)
Hypertension status	Normal BP	633 (85.43)	5124 (81.89)	**0.017**	Reference
	Elevated BP	108 (14.57)	1133 (18.11)		0.79 (0.61-1.03)
Cholesterol status	Normal blood cholesterol	714 (96.75)	6042 (96.90)	0.817	Not included into the primary model
	Raised blood cholesterol	24 (3.25)	193 (3.10)		
Diabetic status	Absence of diabetes	605 (93.51)	5703 (95.34)	0.104	Not included into the primary model
	Impaired fasting glycaemia	13 (2.01)	97 (1.62)		
	Raised fasting blood glucose	29 (4.48)	182 (3.04)		
HIV status*	Positive	33 (5.78)	195 (3.94)	**0.037**	Reference
	Negative	538 (94.22)	4753 (96.06)		**1.64 (1.09-2.47)**
**Behavioral risk factors for CKD**					
Tobacco use during the past 12 months	No	117 (15.79)	906 (14.50)	0.346	Reference
	Yes	624 (84.21)	5344 (85.50)		1.02 (0.77-1.35)
Alcohol consumption during the past 30 days	Yes	372 (90.51)	2593 (88.92)	0.333	Not included into the primary model
	No	39 (9.49)	323 (11.08)		
Number of serving of fruit per day	1	361 (78.31)	3150 (78.59)	0.988	Not included into the primary model
	2	88 (19.09)	753 (18.79)		
	3 and over	12 (2.60)	105 (2.62)		
Number of serving of vegetables per day	1	357 (50.14)	2878 (48.29)	0.545	Reference
	2	340 (47.75)	2971 (49.85)		0.88 (0.73-1.06)
	3 and over	15 (2.11)	111 (1.86)		0.612 (0.64-2.14)
Number of days in a week with moderate physical activity	1	21 (5.57)	149 (5.17)	**< 0.001**	Not included into the primary model
	2	36 (9.55)	339 (11.76)		
	3	41 (10.88)	402 (13.94)		
	4	19 (5.04)	220 (7.63)		
	5	33 (8.75)	415 (14.39)		
	6	130 (34.48)	898 (31.15)		
	7	97 (25.73)	460 (15.96)		

Furthermore, additional analyses explored the association between selected socio-demographic, biological and behavioral variables and are described in the Supplementary materials.

### Multivariable assessment of risk factors associated with albuminuria

All socio-demographic variables were included into the (adjusted) primary model. Among biological and behavioural variables, vegetable consumption, tobacco use and hypertension were retained in the multivariable model as they showed a greater data availability while variables found collinear with these factors (fruit consumption, physical activity, alcohol consumption, blood glucose, cholesterol) were excluded.

Following multivariable adjustment, semi-urban and urban residency were associated with lower odds of albuminuria (OR 0.36, CI 0.23-0.56, *p*<0.001 and OR 0.34, CI 0.23-0.50, *p*<0.001, respectively) than rural status. Additionally, being married or living with a partner had higher odds of albuminuria than being single, with ORs of 1.44 (CI 1.03-2.02, *p*=0.031) and 1.62 (CI 1.06-2.48, *p*=0.026), respectively. Sex of the participant, age group, educational and employment status were not associated with the odds of prevalent albuminuria and tobacco use and vegetable consumption were also not associated with the condition.

Among biological variables, odds of albuminuria were greater among participants living with HIV (OR 1.64, CI 1.09- 2.47, *p*=0.018), while BMI and hypertension status showed no association with the condition (Table 2).

In secondary analyses, diabetic status was not associated with albuminuria (Supplementary materials, model 2).

Furthermore, we grouped the participants of this study according to their blood pressure [54] and assessed their respective odds for developing albuminuria. Results of this secondary analysis showed no significant association between elevated blood pressure and albuminuria and are shown in Supplementary materials (model 3).

## Discussion

We estimated an albuminuria prevalence of 10.59 % (95% CI 9.98-11.33), or 105.9 in 1,000 people in Rwanda. Rural or semi-urban residency category, being married or living with a partner and HIV positivity were significantly associated with higher odds of albuminuria. However, socio-demographic variables such as age and sex as well as risk factors such as tobacco use and vegetable consumption; and biological variables like increased BMI and elevated blood pressure were not associated with albuminuria.

This study reports an albuminuria prevalence of 10.59% (95% CI 9.98-11.33). Wyatt et al. (2011) estimated the prevalence of proteinuria at 8.7% among Rwandan HIV-negative women [33]. This latter is, to the best of our knowledge, one of the few published studies reporting a proteinuria prevalence in Rwanda. The higher prevalence reported in the current study may be attributed to the population-based approach, which included a more diverse study population than the female-only population in Wyatt et al. [33]. In broader populations, similar to the current study, a multisite survey conducted in four countries in Southern, Western and Eastern Africa, found an overall albuminuria prevalence of 9.2% (95% CI 8·4–10·0). However, albuminuria prevalence was reported to vary regionally according to differences socioeconomic status in sub-Saharan Africa [55], potentially explaining a higher overall CKD prevalence of 13.9% (95% CI 12·2–15·7) in a meta-analysis on CKD prevalence which gathered 90 studies in sub-Saharan Africa in 2014 [30]. The high albuminuria prevalence reported in our study and corroborated in other studies from across Africa suggest the need for increased CKD prevention measures in Rwanda, particularly since patients with CKD in resource-constraint countries do not currently have access to hemodialysis or renal replacement therapies [56].

Residency status was identified as risk factor for albuminuria in the current study. Compared with residing in a rural location, living in an urban or semi-urban area indeed indicated a protective effect against albuminuria. This results could be unexpected, as urban lifestyles have been associated with unhealthy lifestyle behaviors such as sedentarity or obesity [57–59]. They might be partly explained by disparities in accessing health care.

Studies conducted in Thailand, China and Cameroon indeed reported limited access to sanitation and health care [60], lower socioeconomic status and health literacy [37, 61] and greater use of herbal medicine and street medications [62] in rural areas compared with urban zones. Therefore, in the area of the Sustainable Development Goals (SDGs) [2] and the motto of leaving no one behind, the Social Determinants of Health (SDH) [63]; should be addressed in a comprehensive manner across all sectors, including inequities in terms of gender and access to health care. Indeed, although Rwanda figures among the few countries in sub-Saharan Africa with government funding for some eligible patients with end-stage renal disease [64]; financial barriers, mainly linked to the high cost of treatment [56], still limit access to renal replacement therapy. A stronger implementation of the WHO’s Health in All Policy framework [65], such as policies aiming at reducing poverty and environmental pollution; and raising awareness on CKD among school-aged population, could thus be beneficial to tackle this disease.

Moreover, being married or living in a union was associated with greater odd of developing albuminuria. This corroborates findings from Bangladesh, where married participants showed a greater tendency to develop CKD than unmarried study subjects [66]. This might be due to an increase in risk factors for NCDs among partnered persons, possibly because partnered persons may be more likely to be older than non-partnered participants, as described in our study, which indicated a higher proportion of older age groups and tobacco and alcohol consumption among married persons; or more likelihood of sedentary lifestyle. For example, married men in Germany and the United States have been reported to have an increased BMI and less physical activity [67, 68]. Similarly, increased BMI was more represented among married participants in our study. The proportion of participants who had a higher moderate physical activity was however greater among married persons, but the lower response rate (less than 50%) associated with this variable might have influenced the results. Additionally, a study conducted in Kenya found higher odds (1.58) of NCD risk factors among married women, but not men [69]. On the other hand, divorced women tended to report worse health than married women in Australia, while no association was found between marital status and health for men [70]. The later could be partly explained by greater shared economic resources among married people compared to other marital statuses and access to a spouse’s health insurance, especially for women [71, 72]. However, a South African study found no association between marital status and NCDs [73].; while married and separated/ divorced/ widower truck drivers in Ethiopia were respectively over three times and two times more at risk of developing NCDs [74]. Moreover, duration of partnership, as well as the bias of self-reporting on partnership considering social acceptability and expectation are other factors that may be further explored in view of the association reported.

Despite the associations between socioeconomic status and partner status and albuminuria reported in the current study, further sociodemographic variables such as age group and gender were not associated with this risk factor for CKD. CKD prevalence in the elderly is usually higher than in the general population, mainly due to changes in the structure of the kidneys [75]. Absence of association between age and albuminuria may be explained by the relatively young age of the participants of this study, as well as survival bias. Participants with albuminuria may indeed have already died at the time of this study compared with subjects with healthy kidneys resulting in a higher representation of non-CKD persons among the older participants [76]. This is sadly the case in settings with limited access to treatment, especially among the poorest populations [37].

In terms of gender, Zhang et al. (2008) similarly reported little difference in CKD prevalence between men and women China [77]. However, differences in CKD risk between genders have been reported across multiple populations, including China [78], US [79], Tanzania [80] and France [81], with females more commonly reported at increased odds . These associations are however complex, and need to account for other factors, such as gender variations in body size [82]-which influences the measurement of the renal function; and socioeconomic disparities, which also influence the sex distribution of the disease [83]. After having accounted for body size through BMI and for socioeconomic factors such as education and employment; this study found that gender was not independently associated with CKD.

Similarly, lifestyle factors such as smoking showed no statistically significant association with albuminuria. Although smoking is known as a risk factor for cardiovascular disease, its role as independent risk factor for CKD is less apparent [84]. Heavy smoking status is indeed directly linked with CKD [85, 86], as demonstrated in the United States [87] and in Syria [86]. The absence of information on the daily use of tobacco products in this research could partly explain the absence of association between smoking and albuminuria in the current study. Furthermore, results from the 2013 STEPS survey indicate that smoking prevalence increases with age in Rwanda [34]. The relatively low [15–64] age range of the subjects may have masked a possible association between smoking and albuminuria in this study. Moreover, as smoking is associated with both greater odds of dying and development and progression of CKD [86, 88], survival bias [89, 90] could thus explain the absence of association between smoking and CKD. Additionally, we found no relationship between vegetable consumption and albuminuria. Although increased vegetable consumption has been reported to lower the mortality from all causes and NCDs, particularly cardio-vascular diseases [91–93]; less evidence exists on the direct effect of vegetable consumption on CKD [94]. A Korean cohort study indicated a protective effect of high vegetable consumption for the renal function compared to low vegetable regimen [95]. However, there was limited scope to understand the association between high and low vegetable consumption with CKD in the current study, with the majority (99%) of participants in the current study consuming between one and two portions of vegetables per day and no available questionnaire option to indicate an absence of vegetable consumption.

Although lifestyle factors were not associated with albuminuria in the current study, HIV positive status was significantly associated with greater odds of prevalent albuminuria. This corroborates results from a systematic review conducted on the African continent, which indicated that HIV infection was a key risk factor for CKD, especially in contexts with higher HIV prevalence [31]. CKD has been reported in between 3.5 to 48.5% of HIV cases, particularly among young adults of African descent [96, 97]. The association between HIV and CKD may be driven by two distinct pathways. Firstly, HIV positivity may be associated with decreased renal function [98] and CKD, mainly due to the local infection of renal cells by the virus [99, 100]. Secondly, long-term nephrotoxicity of antiretroviral drugs may drive this association [101], with a prospective international cohort study reporting that antiretrovirals, especially associations of tenofovir and atazanavir, were associated with CKD [102]. As HIV was found to be a main contributor to the risk of CKD, the renal function should thus be monitored among HIV patients [103]. Additionally, national treatments HIV guidelines may be amended according to the current evidence, especially regarding the monitoring of renal function and the use of certain combinations of antiretrovirals.

HIV was the only biological factor measured in the current study that associated with albuminuria. There was no association between elevated blood pressure and CKD. Hypertension figures among the well-known risk factors for CKD in the United States [104]. The absence of association between hypertension and albuminuria in this study could be due to antihypertensive drugs taken by the participants. However, this information was lacking for over 99% of the study participants. Furthermore, our study found no association between increased BMI and CKD. This is similar with findings from a study using data of the descendants of the Framingham Heart Study in the United States; which indicated no association between overweight or obesity and CKD incidence [105].

This population-based survey followed a robust cross-sectional survey design (STEPS) and allowed the estimation of the national estimate of the prevalence of albuminuria as a key risk factor for CKD in Rwanda, which had been sparsely documented. The STEPS methodology, developed according to current evidence and best practices, permitted to collect and analyze the data in a standardized manner, allowing comparison over time and between countries. In addition, the large sample size and detailed data collection allowed for the exploration of risk factors for albuminuria [106]. Furthermore, our study brought additional evidence on albuminuria prevalence in Rwanda, and factors that may influence its prevalence. Indeed, no nationally representative studies on albuminuria prevalence had been published prior to this work, which generated an accurate estimate of the albuminuria prevalence in Rwanda. Our findings, such as association between residence status and albuminuria, may also be generalizable to developing countries, where non-traditional risk factors for CKD such as the use of herbal/traditional medicine, are more common [60, 62].

The initial STEPS survey was not specifically designed to assess for the prevalence of albuminuria as a key risk factor for CKD but rather to estimate the prevalence of main risk factors related to NCDs in general using a cross-sectional design. The prevalence of albuminuria was therefore assessed using a single measurement, which is a more practical way of screening for CKD than GFR estimation techniques, although it may lead to an overestimation of its prevalence, given the daily variability of albuminuria [107–109] and the possible occurrence of other situations and conditions such as menstrual contamination, urinary infection, temporary illnesses, and strenuous exercise, among others; that could result in transient albuminuria [12]. The presence and severity of CKD can indeed be measured using several methods including: markers of exogenous filtration or blood markers and various GFR-estimating equations indeed exist to assess the GFR [110, 111]; while urinary albumin to creatinine ratio or urine protein dipstick tests measure albuminuria [111, 112]. However, although repeated complex measurements may improve the accuracy of results; their use can challenge the daily clinical practice [111], especially in developing countries, which often lack the resources, skilled laboratory workforce and quality laboratory equipment to perform such tests [113]. Therefore, a single measurement of albuminuria could be cost saving given its discriminative value as a marker of kidney damage [114]. Affordable point of care urine dipstick tests are indeed useful for albuminuria screening and have indicated a high negative predictive value [115, 116]. However, high false-positive rates associated with such tests require laboratory confirmation [116], which was conducted during this study; and study participants with abnormal test results were then referred to the nearest health facility for further investigations and assessment of their renal condition [46]. This follows the KDIGO protocol for individuals demonstrating a positive reagent strip test, which recommends laboratory confirmation of persistent albuminuria on at least two additional occasions, which could also help to exclude the other possible causes of transient albuminuria [12].

Some factors specially related to CKD, such as the use of drugs, exposure to heavy metals and genetics [117] may have thus been omitted. Moreover, associations could not be interpreted as causal due to the cross-sectional nature of the study [106]. Additionally, the questionnaire included self-reported data. Recall biases may thus have occurred, masking some association between the independent variables and albuminuria [118]. Furthermore, this population-based survey included more women (62.82%) than men for all age groups, while the 2012 census indicated that women aged 15-64 years accounted for 51.84% of the population in Rwanda [119]. A possible explanation may be that women were more likely to be present at home during the data collection which took place during the farming period [34]. However, we didn’t find any independent gender differences during this study. Additionally, albuminuria was used as the sole marker of the renal function as per WHO’s STEPS guidelines [44]. However, the KDIGO guidelines recommends urine albumin-to-creatinine ratio (ACR); and GFR equation to evaluate the glomerular filtration rate [12]. Our study used urine dipsticks as first assessment of the renal function because they were relatively inexpensive and more practical in community screening, with a high negative predictive value and minimal risk of a missed diagnosis of CKD [12]. We also conducted laboratory confirmation for positive cases as per international recommendations [12, 116]. However, results from the comparison of dipstick and laboratory tests were not available for this secondary analysis.

## Conclusion

Our study reports a prevalence of albuminuria of 10.59% in Rwanda. Participants residing in rural areas, partnered persons and HIV positive individuals had greater associations with this condition, which is a marker of kidney damage and CKD progression. Improved screening among most-at-risk individuals, including early screening for CKD among the HIV patients is thus needed. However, as CKD is influenced by other social determinants for health, the role of additional causes such as malnutrition and environmental factors, e.g. exposure to nephrotoxic drugs or agents, should be further explored to ensure that CKD doesn’t remain a “neglected chronic disease”.

## Supplementary Information

**Additional file 1.**

## Data Availability

The data that support the findings of this study are available from the Rwanda Biomedical Center but restrictions apply to the availability of these data, which were used under written ethical clearance for the current study, and so are not publicly available. Data are however available from the authors (Dr. Candide Tran Ngoc) upon reasonable request and with permission of the Rwanda Biomedical Center.

## Abbreviations

NCDs: Non-communicable diseases
CKD: Chronic kidney disease
STEPS: STEPwise approach to NCD risk factor Surveillance
IQR: Interquartile range
OR: Odds ratio
HIV: Human immunodeficiency virus
BMI: Body mass index
GFR: Glomerular filtration rate
ESRD: End-stage renal disease
WHO: World Health Organization
EAs: Enumeration areas
RNEC: Rwanda National Ethics Committee
PDAs: Personal digital assistants
ACR: Albumin-to-creatinine ratio
