# Supplementary material for: Risk factors associated with albuminuria in Rwanda: results from a STEPS survey
Source: BMC Nephrol. 2021 Nov 1;22:361. doi: 10.1186/s12882-021-02574-w (PMC8561895; doi:10.1186/s12882-021-02574-w)
Supplement: Supplementary file 1 — Additional file 1. [file 12882_2021_2574_MOESM1_ESM.docx]

**Additional file 1**

1. **Proportion of selected socio-demographic, biological and behavioral characteristics according to marital status and residence**

|  |  | **Marital status  N (%)** | | | | | |
| --- | --- | --- | --- | --- | --- | --- | --- |
| **Selected variables** | **Variable**  **category** | Single | Cohabitating | Currently married | Separated | Divorced | Widowed |
| **Age group** | 15-24 | 1,088  (74.52%) | 104 (7.12%) | 248  (16.99%) | 13 (0.89%) | 4  (0.27%) | 3  (0.21%) |
|  | 25-34 | 393 (17.09%) | 222 (9.66%) | 1,539 (66.94%) | 65  (2.83%) | 53  (2.31%) | 27 (1.17%) |
|  | 35-44 | 113  (7.47%) | 105  (6.94%) | 1,070 (70.77%) | 57  (3.77%) | 47  (3.11%) | 105  (6.94%) |
|  | 45-54 | 41  (4.02%) | 44  (4.32%) | 696 (68.30%) | 56  (5.50%) | 22  (2.16%) | 160  (15.70%) |
|  | 55-64 | 12  (1.73%) | 18  (2.60%) | 420  (60.69%) | 35  (5.06%) | 17  (2.46%) | 190  (27.46%) |
| **BMI** | Normal | 1,266  (24.38%) | 382  (7.36%) | 2,917 (56.18%) | 161  (3.10%) | 92 (1.77%) | 374  (7.20%) |
|  | Underweight | 162  (31.33%) | 20  (3.87%) | 255  (49.32%) | 21  (4.06%) | 18  (3.48%) | 41  (7.93%) |
|  | Overweight | 182  (17.91%) | 75  (7.38%) | 639  (62.89%) | 34  (3.35%) | 25  (2.46%) | 61  (6.00%) |
|  | Obese | 29  (12.03%) | 16  (6.64%) | 157  (65.15%) | 8  (3.32%) | 6  (2.49%) | 25  (10.37%) |
| **Tobacco use in the past 12 months** | No | 1,527 (25.61%) | 398  (6.67%) | 3,368  (56.48%) | 184  (3.09%) | 112 (1.88%) | 374  (6.27%) |
|  | Yes | 119  (11.64%) | 95  (9.30%) | 608  (59.49%) | 42  (4.11%) | 31  (3.03%) | 127  (12.43%) |
| **Alcohol use in the past 30 days** | No | 101 (27.90%) | 35 (9.67%) | 168 (46.41%) | 21 (5.80%) | 7 (1.93%) | 30 (8.29%) |
|  | Yes | 533 (17.98%) | 264  (8.91%) | 1,791 (60.43%) | 99 (3.34%) | 66 (2.23%) | 211 (7.12%) |
| **Number of serving of fruit per day** | 1 | 910  (25.95%) | 213  (6.07%) | 2,014 (54.82%) | 99 (2.82%) | 69 (1.97%) | 202 (5.76%) |
|  | 2 | 234  (27.82%) | 59 (7.02%) | 461 (54.82%) | 22 (2.62%) | 15 (1.78%) | 50 (5.95%) |
|  | 3 and over | 38 (32.48%) | 6 (5.13%) | 66 (56.41%) | 1 (0.85%) | 3 (2.56%) | 3 (2.56%) |
| **Number of serving of vegetables per day** | 1 | 828  (25.60%) | 241  (7.45%) | 1,776  (54.92%) | 98  (3.03%) | 66  (2.04%) | 225  (6.96%) |
|  | 2 | 712  (21.54%) | 216  (6.53%) | 1,948 (58.92%) | 116  (3.51%) | 64  (1.94%) | 250  (7.56%) |
|  | 3 and over | 31  (24.60%) | 6  (4.76%) | 80  (63.49%) | 1  (0.79%) | 3  (2.38%) | 5  (3.97%) |
| **Number of days in a week with moderate physical activity** | 1 | 57 (33.53%) | 9 (5.29%) | 90  (52.94%) | 3 (1.76%) | 2 (1.18%) | 9 (5.29%) |
|  | 2 | 105 (28.07%) | 22 (5.88%) | 212 (56.68%) | 9 (2.41%) | 10 (2.67%) | 16 (4.28%) |
|  | 3 | 124 (27.99%) | 37 (8.35%) | 229 (51.69%) | 10 (2.26%) | 10 (2.26%) | 33 (7.45%) |
|  | 4 | 60 (25.10%) | 22 (9.21%) | 134 (56.07%) | 4 (1.67%) | 2 (0.84%) | 17 (7.11%) |
|  | 5 | 107 (23.88%) | 42 (9.38%) | 242 (54.02%) | 15 (3.35%) | 8 (1.79%) | 34 (7.59%) |
|  | 6 | 142 (13.83%) | 124 (12.07%) | 593 (57.74%) | 52 (5.06%) | 24 (2.34%) | 92 (8.96%) |
|  | 7 | 142 (25.49%) | 21 (3.77%) | 318 (57.09%) | 16 (2.87%) | 17 (3.05%) | 43 (7.72%) |
|  |  |  |  |  |  |  |  |

|  |  | **Residence N (%)** | | |
| --- | --- | --- | --- | --- |
| **Selected variables** | **Variable category** | Rural | Urban | Semi-urban |
| **Age group** | 15-24 | 1,077  (73.77%) | 262  (17.95%) | 121  (8.29%) |
|  | 25-34 | 1,791 (77.67%) | 304 (13.18%) | 211 (9.15%) |
|  | 35-44 | 1,208 (79.84%) | 185 (12.23%) | 120 (7.93%) |
|  | 45-54 | 842  (82.55%) | 100 (9.80%) | 78 (7.65%) |
|  | 55-64 | 569 (81.99%) | 69 (9.94%) | 56 (8.07%) |
| **BMI** | Normal | 4,240 (81.51%) | 543 (10.44%) | 419 (8.05%) |
|  | Underweight | 406 (78.38%) | 61 (11.78%) | 51 (9.85%) |
|  | Overweight | 734 (72.17%) | 187 (18.39%) | 96 (9.44%) |
|  | Obese | 95 (39.42%) | 127 (52.70%) | 19 (7.88%) |
| **Tobacco use in the past 12 months** | No | 4,641 (77.76%) | 823 (13.79%) | 504 (8.45%) |
|  | Yes | 842 (82.31%) | 98 (9.58%) | 83 (8.11%) |
| **Alcohol use in the past 30 days** | No | 271 (74.86%) | 51 (14.09%) | 40 (11.05%) |
|  | Yes | 2,419 (81.59%) | 283 (9.54%) | 263 (8.87%) |
| **Number of serving of fruit per day** | 1 | 2,684 (76.45%) | 542 (15.44%) | 285 (8.12%) |
|  | 2 | 653 (77.65%) | 113 (13.44%) | 75 (8.92%) |
|  | 3 and over | 88 (75.21%) | 16 (13.68%) | 13 (11.11%) |
| **Number of serving of vegetables per day** | 1 | 2,578 (79.69%) | 391 (14.32%) | 266 (8.82%) |
|  | 2 | 2,545 (76.86%) | 474 (53.74%) | 292 (51.59%) |
|  | 3 and over | 101 (80.16%) | 17 (13.49%) | 8 (6.35%) |
| **Number of days in a week with moderate physical activity** | 1 | 141 (82.94%) | 18 (10.59%) | 11 (6.47%) |
|  | 2 | 274  (73.07%) | 58 (15.47%) | 43 (11.47%) |
|  | 3 | 331 (74.72%) | 42 (9.48%) | 70  (15.80%) |
|  | 4 | 182 (76.15%) | 28 (11.72%) | 29 (12.13%) |
|  | 5 | 356 (79.46%) | 37 (8.26%) | 55 (12.28) |
|  | 6 | 912 (88.72%) | 47 (4.57%) | 69 (6.71%) |
|  | 7 | 441  (79.17%) | 81 (14.54%) | 35 (6.28%) |

1. **Results of the Spearman’s correlation test for continuous variables**

| **Compared variables** | **Spearman's coefficient (Rho)** | **p-value** |
| --- | --- | --- |
| Vegetable consumption and fruit consumption | 0.2273 | <0.0001 |
| Vegetable consumption and physical activity | 0.1027 | <0.001 |
| Vegetable consumption and BMI | -0.0071 | 0.5563 |
| Physical activity and BMI | -0.0141 | 0.4161 |
| Physical activity and blood pressure (SYST) | -0.0154 | 0.3746 |
| Physical activity and blood pressure (DIAST) | -0.0036 | 0.8362 |
| Physical activity and cholesterol | 0.0326 | 0.0619 |
| Physical activity and blood glucose | -0.0189 | 0.2933 |
| Physical activity and daily number of smoking products used | -0.0451 | 0.3947 |
| Alcohol units and vegetable | -0.0348 | 0.0643 |
| Alcohol units and daily number of smoking products used | 0.2557 | <0.0001 |
| Alcohol and blood glucose | 0.0165 | 0.3895 |
| Alcohol and cholesterol levels | 0.0014 | 0.9403 |
| Blood glucose and cholesterol | 0.172 | <0.0001 |
| Blood glucose and blood pressure (SYST) | 0.0481 | 0.0001 |
| Blood glucose and blood pressure (DIAST) | 0.0434 | 0.0004 |
| Cholesterol and blood pressure (SYST) | 0.0923 | <0.0001 |
| Cholesterol and blood pressure (DIAST) | 0.1323 | <0.0001 |

1. **Secondary models**

**Model 2**

Number of observations: 4,973 (71% of participants)

| **Variable** | **odds ratio (OR)** | **95% confidence interval (CI)** | **p-value** |
| --- | --- | --- | --- |
| **Sex** |  |  |  |
| Male | 1 |  |  |
| Female |  | 0.76-1.17 | 0.591 |
| **Age group** |  |  |  |
| 15-24 | 1 |  |  |
| 25-34 |  | 0.60- 1.23 | 0.410 |
| 35-44 |  | 0.84- 1.75 | 0.313 |
| 45-54 |  | 0.55- 1.29 | 0.431 |
| 55-64 |  | 0.53- 1.44 | 0.593 |
| **Residence** |  |  |  |
| Rural | 1 |  |  |
| Semi urban |  | 0.22-0.57 | <0.001 |
| Urban |  | 0.24- 0.54 | <0.001 |
| **Education** |  |  |  |
| No formal education | 1 |  |  |
| Completed primary |  | 0.86- 1.30 | 0.623 |
| Completed secondary |  | 0.47- 1.76 | 0.776 |
| Completed at least undergraduate university |  | 0.33- 2.47 | 0.838 |
| **Employment status** |  |  |  |
| Employee | 1 |  |  |
| Self-employed |  | 0.50- 1.76 | 0.847 |
| Non-paid work |  | 0.50- 2.65 | 0.750 |
| Student |  | 0.47- 2.60 | 0.814 |
| Retired |  | 0.38- 43.00 | 0.250 |
| Unemployed |  | 0.82- 3.53 | 0.152 |
| **Marital status** |  |  |  |
| Single | 1 |  |  |
| Currently married |  | 1.02- 2.09 | 0.039 |
| Separated |  | 0.58- 2.21 | 0.716 |
| Divorced |  | 0.86- 3.51 | 0.126 |
| Widowed |  | 0.71- 2.11 | 0.465 |
| Cohabitating |  | 0.98- 2.46 | 0.059 |
| **Current smoking status** |  |  |  |
| No | 1 |  |  |
| Yes |  | 0.79- 1.42 | 0.716 |
| **Number of servings of vegetables per day** |  |  |  |
| 1 | 1 |  |  |
| 2 |  | 0.76- 1.12 | 0.400 |
| 3 and over |  | 0.69- 2.44 | 0.409 |
| **BMI** |  |  |  |
| Normal | 1 |  |  |
| Underweight |  | 0.76- 1.63 | 0.593 |
| Overweight |  | 0.62- 1.10 | 0.183 |
| Obese |  | 0.68- 2.01 | 0.561 |
| **Diabetic status** |  |  |  |
| Absence of diabetes (capillary blood glucose <5.6 mmol/L) | 1 |  |  |
| Impaired fasting glycaemia (capillary blood glucose >=5.6mmol/L and <6.1 mmol/L) |  | 0.97- 3.28 | 0.061 |
| Raised fasting blood glucose (capillary blood glucose >=6.1 mmol/L) |  | 0.96- 2.61 | 0.074 |
| **HIV status** |  |  |  |
| Negative | 1 |  |  |
| Positive |  | 1.15- 2.67 | 0.010 |

**Model 3**

4,897 participants (70.0% of participants)

| **Variable** | **odds ratio (OR)** | **95% confidence interval (CI)** | **p-value** |
| --- | --- | --- | --- |
| **Sex** |  |  |  |
| Male | 1 |  |  |
| Female | 0.94 | 0.76-1.16 | 0.569 |
| **Age group** |  |  |  |
| 15-24 | 1 |  |  |
| 25-34 | 0.86 | 0.60-1.22 | 0.396 |
| 35-44 | 1.21 | 0.84-1.76 | 0.308 |
| 45-54 | 0.88 | 0.57-1.35 | 0.55 |
| 55-64 | 0.99 | 0.59-1.65 | 0.963 |
| **Residence** |  |  |  |
| Rural | 1 |  |  |
| Semi urban | 0.36 | 0.22-0.58 | <0.001 |
| Urban | 0.36 | 0.24-0.54 | <0.001 |
| **Education** |  |  |  |
| No formal education | 1 |  |  |
| Completed primary | 1.06 | 0.86-1.31 | 0.579 |
| Completed secondary | 0.92 | 0.47-1.78 | 0.800 |
| Completed at least undergraduate university | 0.89 | 0.32-2.46 | 0.829 |
| **Employment status** |  |  |  |
| Employee | 1 |  |  |
| Self-employed | 0.93 | 0.49-1.76 | 0.832 |
| Non-paid work | 1.14 | 0.49-2.65 | 0.759 |
| Student | 1.08 | 0.46-2.53 | 0.868 |
| Retired | 4.45 | 0.40-50.0 | 0.226 |
| Unemployed | 1.69 | 0.80-3.47 | 0.171 |
| **Marital status** |  |  |  |
| Single | 1 |  |  |
| Currently married | 1.45 | 1.01-2.08 | 0.042 |
| Separated | 1.14 | 0.58-2.22 | 0.704 |
| Divorced | 1.7 | 0.84-3.45 | 0.14 |
| Widowed | 1.14 | 0.65-1.97 | 0.652 |
| Cohabitating | 1.56 | 0.99-2.47 | 0.057 |
| **BMI** |  |  |  |
| Normal blood pressure | 1 |  |  |
| Underweight | 1.06 | 0.72- 1.57 | 0.767 |
| Overweight | 0.82 | 0.62- 1.10 | 1.10 |
| Obese | 1.19 | 0.70- 2.05 | 0.524 |
| **Hypertension status** |  |  |  |
| Normal BP (syst <140 and diast <90) | 1 |  |  |
| Grade 1 HTA (syst >=140 and =< 159 or diast >=90 and =<99) | 0.86 | 0.61-1.12 | 0.266 |
| Grade 2 HTA (syst >=160 or diast >=100) | 0.27 | 0.06-1.11 | 0.070 |
| **Cholesterol status** |  |  |  |
| Normal | 1 |  |  |
| Raised blood cholesterol | 1.14 | 0.64-2.00 | 0.66 |
| **Diabetic status** |  |  |  |
| Normal | 1 |  |  |
| Impaired fasting glycaemia | 1.80 | 0.98-3.32 | 0.058 |
| Raised blood fasting glucose | 1.64 | 0.98-2.74 | 0.057 |
| **HIV status** |  |  |  |
| Negative | 1 |  |  |
| Positive | 1.83 | 1.19-2.79 | 0.005 |
| **Current smoking status** |  |  |  |
| No | 1 |  |  |
| Yes | 1.04 | 0.78-1.41 | 0.773 |
| **Number of servings of vegetables per day** |  |  |  |
| 1 | 1 |  |  |
| 2 | 0.93 | 0.76-1.13 | 0.439 |
| 3 and over | 1.33 | 0.70-2.50 | 0.372 |
